# 

**Published:** 1908-04-18

**Authors:** 


### The Keeping Properties of Infusion of Quassia.

E. Quant has recently made experiments with the object of ascertaining the best method of preparing infusion of quassia. He finds that the present official process—that is to say, a cold-water infusion—makes a better product than if boiling water were used. He suggests, however, that the infusion is more stable if, after being made, it is boiled for a few minutes.

### An Improved Form of Liquid Extract of Cascara Sagrada.

The advantages of a miscible liquid extract of cascara sagrada are obvious, and a simple method for preparing such a compound was communicated to the British Pharmaceutical Conference by J. H. Franklin. He suggests the use of glycerin instead of alcohol as a preservative, and the addition to each ounce of the finished product of a few minims of strong solution of ammonia. A liquid extract made in the way suggested does not become cloudy on the addition of water, and the addition of ammonia is stated not to interfere with the therapeutic activity of the preparation.

### The Institute of Social Service.

The British Institute of Social Service is, according to its own definition, "a clearing house of civilisation." In other words, it collects, registers, and disseminates information relating to all forms of social work. Hardly to be called a society in itself, it is the guide-post to all societies, and welcomes inquiries from all who desire information on questions affecting the amelioration of present conditions. There is hardly any difficulty or trouble of life for which we have not means to cope. There are societies, associations, homes, clubs—apparatus for as nearly as possible every form of distress that human nature in a social system such as ours can fall heir to. The trouble is that the sufferer so often does not know where to find the help he needs, and that his friends do not know where to direct him. This is the want that the Institute of Social Service proposes to meet. It exists to answer questions. As every question is tabulated with its answer, the matter of replying grows easier as the inquiries increase in number, though it is very rare that just the same reply can meet two queries. Still, each accumulates information which may be called upon for future use, until by degrees a practical reference library is established. That the Institute is being taken advantage of to find out what apparatus exists for the needs of the poor and afflicted may be inferred from the fact that 472 queries were addressed to it during last year, coming from all parts of the world and dealing with subjects educational, municipal, philanthropic, bibliographical, and many others too various to be detailed. As 3,900 letters have been received and 4,000 written, it may be guessed that the clerical staff has had enough of work, and that the motto of the Institute, "The experience of all for the benefit of each," is being carried out both inside and outside the office, which is to be found at 11 Southampton Row, W.C.

### FORTHCOMING BOOKS.

Messrs. J. and A. Churchill will shortly publish for the May session the eighth edition of "A Manual of the Practice of Medicine," by Dr. Frederick Taylor; the second edition of "A Manual of Ophthalmic Surgery and Medicine," by Mr. W. H. H. Jessop; the fifth edition of "A Short Practice of Midwifery," by Dr. Jellett; and the eighth edition of "Qualitative Analysis and Practical Chemistry," by Dr. Frank Clowes. Also new works, entitled "A Manual for Midwives," by Dr. Longridge, of Queen Charlotte's Hospital, and "The Thermal Treatment of Aix-les-Bains," by Dr. H. Forestier.

THE HOSPITAL

*April* 18, 1908.

*Name* ..............................................................................................

*Address* ..............................................................................................

..............................................................................................

This Coupon must accompany manuscript or contributions intended for The Hospital.

